# A patient-derived xenograft pre-clinical trial reveals treatment responses and a resistance mechanism to karonudib in metastatic melanoma

**DOI:** 10.1038/s41419-018-0865-6

**Published:** 2018-07-24

**Authors:** Berglind O. Einarsdottir, Joakim Karlsson, Elin M. V. Söderberg, Mattias F. Lindberg, Elisa Funck-Brentano, Henrik Jespersen, Siggeir F. Brynjolfsson, Roger Olofsson Bagge, Louise Carstam, Martin Scobie, Tobias Koolmeister, Olof Wallner, Ulrika Stierner, Ulrika Warpman Berglund, Lars Ny, Lisa M. Nilsson, Erik Larsson, Thomas Helleday, Jonas A. Nilsson

**Affiliations:** 1 Sahlgrenska Translational Melanoma Group, Sahlgrenska Cancer Center, Departments of Surgery and Oncology, Institute of Clinical Sciences, University of Gothenburg and Sahlgrenska University Hospital, Gothenburg, Sweden; 2 Department of Medical Chemistry, Institute of Biomedicine, University of Gothenburg, Gothenburg, Sweden; 3 Department of Microbiology and Immunology, Institute for Biomedicine, Sahlgrenska Academy, University of Gothenburg, Gothenburg, Sweden; 4 Department of Neurosurgery, Sahlgrenska University Hospital, Gothenburg, Sweden; 5 Science for Life Laboratory, Division of Translational Medicine and Chemical Biology, Department of Medical Biochemistry and Biophysics, Karolinska Institutet, Stockholm, Sweden

## Abstract

Karonudib (TH1579) is a novel compound that exerts anti-tumor activities and has recently entered phase I clinical testing. The aim of this study was to conduct a pre-clinical trial in patient-derived xenografts to identify the possible biomarkers of response or resistance that could guide inclusion of patients suffering from metastatic melanoma in phase II clinical trials. Patient-derived xenografts from 31 melanoma patients with metastatic disease were treated with karonudib or a vehicle for 18 days. Treatment responses were followed by measuring tumor sizes, and the models were categorized in the response groups. Tumors were harvested and processed for RNA sequencing and protein analysis. To investigate the effect of karonudib on T-cell-mediated anti-tumor activities, tumor-infiltrating T cells were injected in mice carrying autologous tumors and the mice treated with karonudib. We show that karonudib has heterogeneous anti-tumor effect on metastatic melanoma. Thus, based on the treatment responses, we could divide the 31 patient-derived xenografts in three treatment groups: progression group (32%), suppression group (42%), and regression group (26%). Furthermore, we show that karonudib has anti-tumor effect, irrespective of major melanoma driver mutations. Also, we identify high expression of *ABCB1*, which codes for p-gp pumps as a resistance biomarker. Finally, we show that karonudib treatment does not hamper T-cell-mediated anti-tumor responses. These findings can be used to guide future use of karonudib in clinical use with a potential approach as precision medicine.

## Introduction

Cutaneous melanoma is the most aggressive form of skin cancer and is often fatal in metastatic stages^1^. Recent advances in melanoma genetics and how melanoma cells escape immune attack have resulted in the development of targeted therapies inhibiting the MAPK pathway as well as immunotherapies. These treatments have improved the overall survival and sometimes have resulted in patients possibly being cured from their disease^2^. Despite these successes, new treatments are needed, since the majority of patients are still not cured.

The recently developed inhibitor, karonudib (TH1579), was designed to inhibit the oxidized nucleotide-sanitizing enzyme, MTH1 (encoded by *NUDT1*)^3, 4^. The compound has shown promising anti-tumor effect both in vitro and in vivo^4^ and has initiated phase I clinical testing in cancer patients with advanced solid malignancies (NCT03036228). Lately, known anti-cancer effects, such as microtubule inhibition, have been proposed for compounds containing the same scaffold as karonudib (TH588 and TH287) (unpublished observations)^5–7^. The exact mechanism of action of karonudib and the possible role of MTH1 in cancer is therefore under investigation.

Regardless of the mechanism of action of the compound, karonudib exhibits good cytotoxic effect in cancer cells both in vitro and in vivo, while being well tolerated in non-transformed cells^3, 4^. However, the extent of inter-patient heterogeneity of the cytotoxic effect has not been determined. Furthermore, no predictive biomarkers of response have been identified. These could be used to identify which patients may benefit the most from this treatment and which patients may be spared. Lastly, given the recent successes of immunotherapies in melanoma, karonudib needs to be assessed for its potential impact on T-cell immunity. Here we made use of our melanoma PDX platform^8^ and a novel immune oncology PDX model^9^ to assess the heterogeneous responses observed when treating metastatic melanoma tumors in multiple patients with karonudib. We show that karonudib has anti-tumor effect in a majority of PDX models, but some PDX models remain unaffected, which in some cases can be attributed to known anti-cancer drug resistance mechanisms. Reassuringly though, we also demonstrate that karonudib treatment does not hamper cytotoxic T-cell anti-tumor activities.

## Results

### Heterogeneous response to karonudib treatment in melanoma PDXes

**Fig. 1:**
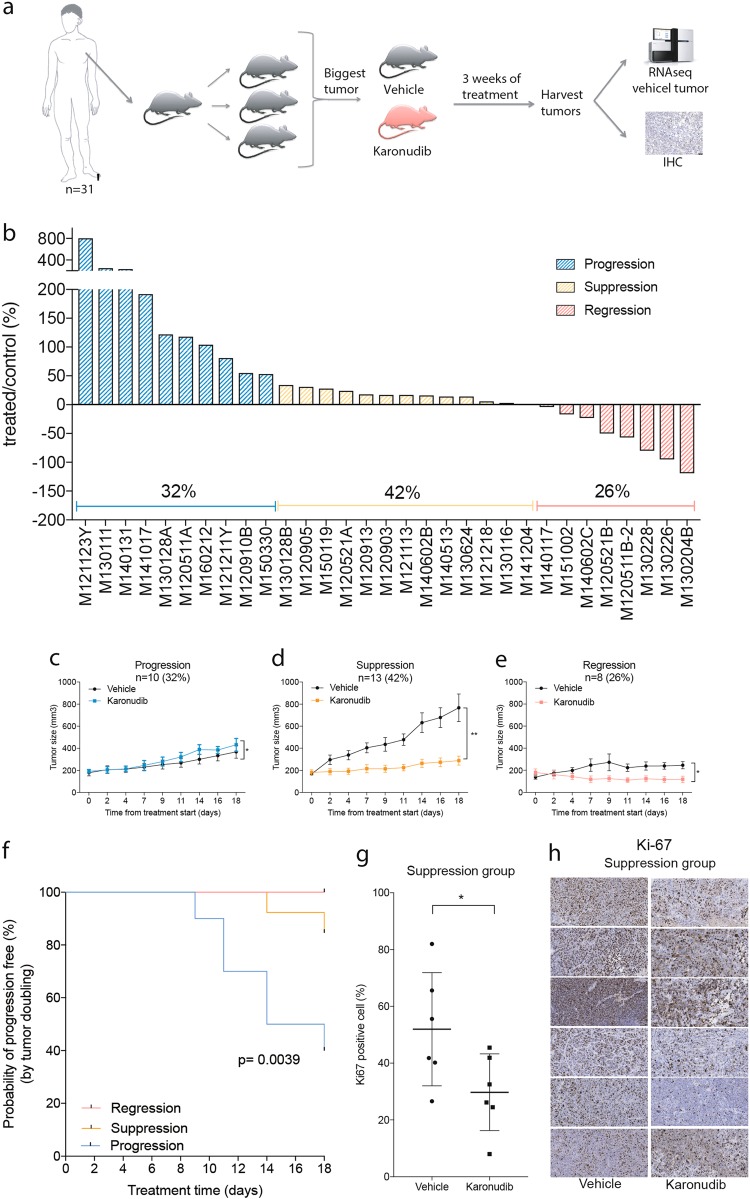
A patient-derived xenograft clinical trial reveals a heterogeneous response to karonudib. **a** Schematic overview of the experimental setup. Tumor biopsy from melanoma patient was serially transplanted twice in mice before being transplanted in mice treated with either karonudib or vehicle. For each patient, there was one mouse per treatment group, which was treated for 18 days before tumors were harvested and processed. Each tumor was snap-frozen for RNA analysis and embedded in paraffin for immunohistochemical straining. **b** Waterfall plot revealing the heterogenous response to karonudib. The samples are divided in three response groups, progression (blue), suppression (yellow), and regression (red). For treatment response catagorization, see Materials and methods. Comparison of average (±SEM) growth speed of karonudib treated vs. vehicle treated PDXes for (**c**) progression group, **d** suppression group, and **e** regression group. Individual growth curves are shown in supplemental figures S2-S4. **f** Kaplan–Meier graph showing the comparison of probability of progression-free survival in karonudib-treated mice (based on tumor doubling time) for the three response groups (see Materials and methods for criterion), Mantel–Cox test used for statistical analysis. **g** Quantification of immunohistochemical staining for the proliferation marker Ki-67 in vehicle vs. karonudib-treated PDXes, Student’s *t* test showing statistically significant difference between the two groups (*p* = 0.0476). **h** Images of the xenograft sections stained with Ki-67 and used for the quantification in (**g**)

We have previously shown that TH588 and karonudib can inhibit the tumor growth of one of our PDX models^3, 4^. However, the heterogeneous responses of metastatic melanoma have not been modeled. To that end, we designed a pre-clinical PDX trial consisting of tumor samples from 31 metastatic melanoma patients representing models of most subtypes (Supplemental table 1). All samples had been serially transplanted in NOG mice as previously described^8^. Each sample was transplanted in three mice, and the two fastest growing xenografts were divided into two treatment groups, thus utilizing one mouse per patient per treatment group (1 × 1 × 1) as previously described^10^. The groups were treated with either 90 mg/kg karonudib or with the vehicle. All mice tolerated the treatment, as evidenced by their stable weights throughout the treatment (Supplemental figures S2-S4). After 3 weeks of treatment, the mice were sacrificed and tumors were harvested. A piece from each tumor was embedded in paraffin for immunohistochemical staining analysis, and a piece of each vehicle-treated tumor was RNA sequenced for mutation and expression analysis (Fig. 1a).

After 3 weeks of treatment, heterogeneous responses to karonudib treatment were observed for the 31 PDX models (Fig. 1b and Supplemental figure S1-S4). Twenty-six percent (8/31) of the PDX models were regressed during karonudib treatment and thus, were categorized as the regression group. Forty-two percent (13/31) of the PDX models responded to karonudib treatment by reduced growth, compared with vehicle-treated tumors, and were categorized as the suppression group. Interestingly though, 32% (10/31) of the PDX models did not exhibit any reduced growth of karonudib-treated tumors, compared with vehicle; these were therefore categorized as the progression group.

To further investigate the treatment responses, the growth of each karonudib-treated PDX was compared with the growth of its matching vehicle-treated PDX (Fig. 1c–e). A significant difference in growth speed of the vehicle-treated PDXes was observed between the response groups, where the fastest growing PDXes were found in the suppression group. Furthermore, statistically significant difference was observed in the probability of progression-free survival (PFS), as based on tumor doubling time between the groups (*p* = 0.0039) (Fig. 1f). This markedly suppressed growth upon karonudib treatment correlated significantly (*p* = 0.03) to lower amount of Ki67 positive cells compared to the vehicle-treated mice in the suppression group, as analyzed with immunohistochemistry (Fig. 1g, h).

### Karonudib has cytotoxic effect on melanoma tumors, irrespective of genotype

**Fig. 2:**
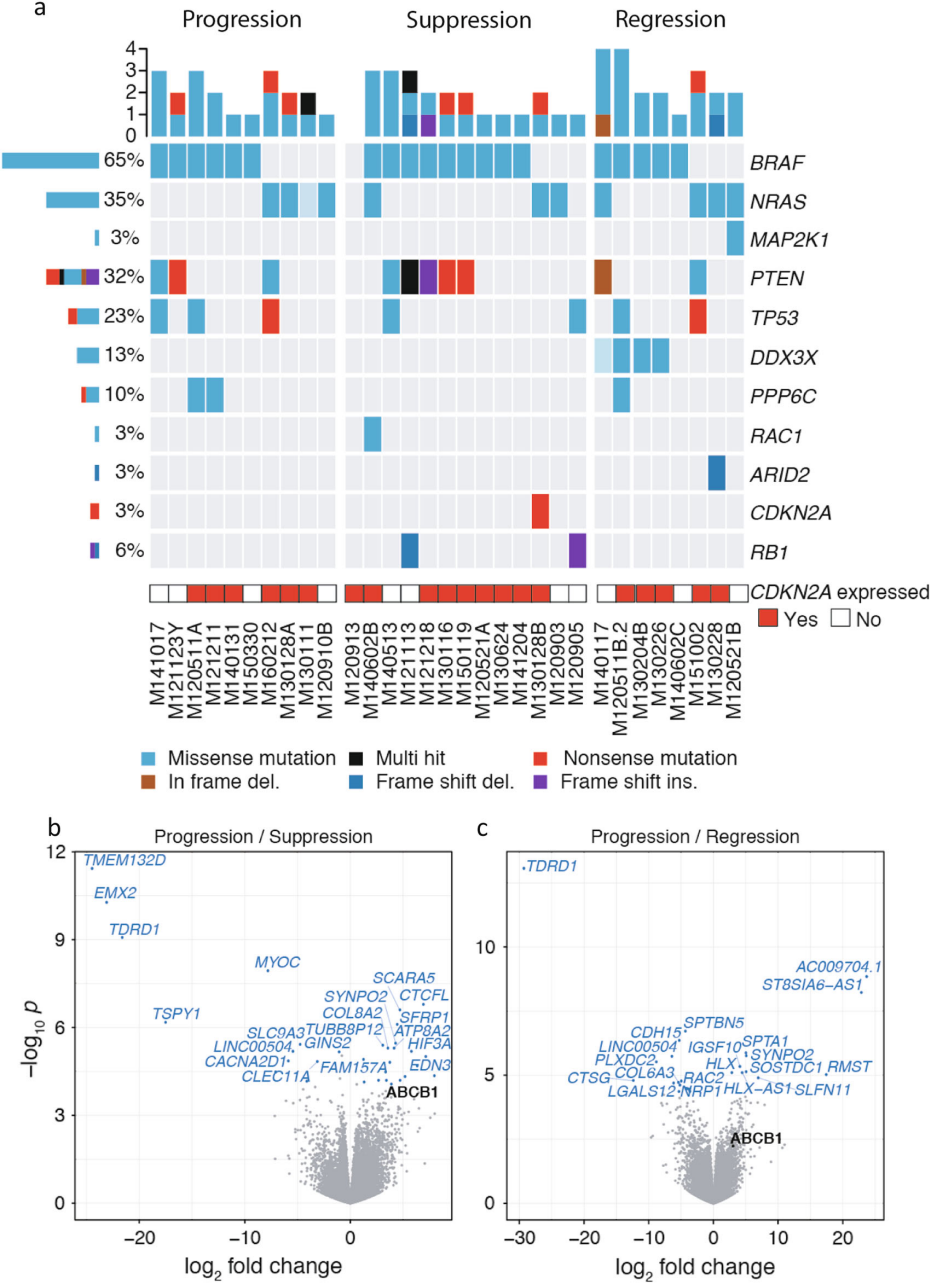
Karonudib has cytotoxic effect, irrespective of melanoma driver mutations. **a** Oncoprint showing melanoma hot-spot mutations found in three response groups. Transparent colors indicate low confidence variants (Materials and methods). **b, c** Volcano plots showing differentially expressed genes between the progression group and (**b**) suppression group, and (**c**) regression group

To investigate if the cytotoxic effect of karonudib was dependent on the genotype, the vehicle tumors were RNA sequenced and the mutation profile of the samples were analyzed. This analysis revealed *BRAF* and *NRAS* mutation in 65% (20/31) and 35% (11/31) of the samples, respectively. Comparison of the mutation profile between the response groups revealed that karonudib has cytotoxic effect in melanoma PDXes, irrespective of the presence of the genotype of the most common driver genes in melanoma, although a trend for an association between mutation in *DDX3X* and the regression group was observed (Fig. 2a and Supplemental figure S5A). However, the mutations in *DDX3X* were difficult to interpret, since these can be spread throughout the coding sequence and could possibly also arise by RNA editing.

Further investigation of the *DDX3X* mutation revealed increased PFS for karonudib-treated PDXes harboring the mutation when compared to the wild-type PDXes, even though not statistically significant (Supplemental figure S5b). Also, three different *DDX3X* missense mutations were revealed (Supplemental figure S5c and Supplemental table S2). To investigate if the impairment of the DDX3X protein had functional relevance when treating tumor cells with karonudib, the melanoma cell line MML-1 was transfected with a siRNA targeted against DDX3X and the cells treated with karonudib. A statistically significant lower expression of *DDX3X* was observed in the siDDX3X -transduced cells compared with the control-transduced cells (*p* = 0.0003; Supplemental figure S5d). *DDX3X* knockdown had a cytotoxic effect, and only 40% of the cells were alive 36 h after transfection (*p* < 0.0001; Supplemental figure S5e). When the DDX3X siRNA-transfected cells were treated with increased concentration of karonudib, a reduced number of live cells was observed when treated with 0.05 µM karonudib compared with the control (adjusted *p* value = 0.0002) (Supplemental figure S5f).

### High expression of ABCB1 identified as a potential resistance biomarker

**Fig. 3:**
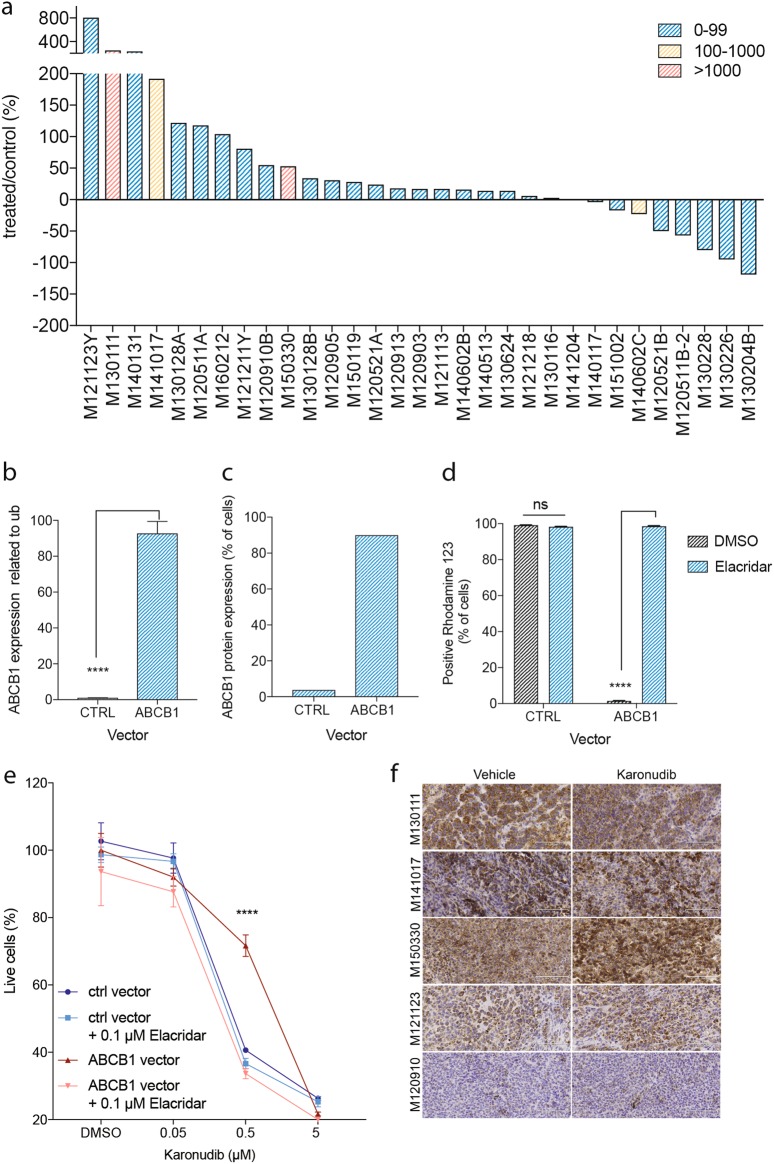
High *ABCB1* expression as a resistance biomarker. **a** A waterfall plot showing treatment response for each of the PDX sample (see criterion in Materials and methods). Bars are color coded according to expression level of *ABCB1* in the vehicle-treated PDXes, blue = 0–99 normalized reads, yellow = 100–1000 normalized reads, and red = >1000 normalized reads (for normalization method used see Materials and methods). **b** Quantitative analysis of *ABCB1* expression in SK-MEL-2 cells after transduction with an *ABCB1*-expressing virus, compared with the control virus (±SD) (*p* < 0.0001), as analyzed with qRT-PCR. **c** Quantitative analysis of protein expression in the same cells, as **b**, analyzed with flow cytometry. **d** Quantitative analysis of activity of p-gp pumps using the p-gp substrate Rhodamine 123, as analyzed using flow cytometry, cells either treated with the p-gp inhibitor Elacridar (1 µM) or DMSO. **e** SK-MEL-2 cells transduced with ABCB1-expressing virus or control virus, treated with different concentrations of karonudib in combination with 0.1 µM Elacridar or DMSO for 48 h. Cells analyzed using flow cytometry, and the data shown as average of triplicate (±SD). Statistically significant rescue effect was observed when SK-MEL-2^ABCB1^ cells were treated with 0.5 µM karonudib (adjusted *p* value 2.6*10^−8^). **f** Immunohistochemical staining of ABCB1 in vehicle and karonudib-treated xenografts from the progression group

To screen for a predictive or a resistance biomarker for karonudib treatment, the association between the gene expression profile of the samples and response groups was investigated. Differential gene expression analysis was performed using the RNA-sequencing data from the vehicle-treated samples (for information regarding total reads per sample, see supplemental table S3). Comparison of the progression group to the suppression and the regression groups revealed differentially expressed genes, one of which was *ABCB1* (Fig. 2b, c and supplemental table S4). Although highly expressed only in two of the samples in the progression group (*p* = 8.52*10^−5^ and *q* = 0.049 compared to the suppression group), it was of interest, since it encodes the multi-drug resistance (MDR) pump, also known as the p-glycoprotein pump (p-gp) (Fig. 3a).

To address the functional importance of high *ABCB1* expression, we transduced the melanoma cell line SK-MEL-2 with either an ABCB1-expressing virus or a control virus. Using quantitative real-time PCR, we observed statistically higher expression of ABCB1 in the ABCB1-transduced cells, compared to the control (*p* < 0.0001) (Fig. 3b). Furthermore, we verified that the high mRNA expression was translated to protein level, using flow cytometry analysis (Fig. 3c). The pumping activity of p-gp pumps can be followed by flow cytometry analysis of the p-gp substrate Rhodamine 123. Cells with functional pumps cultured in the presence of the substrate will pump out Rhodamine 123 and become negative for the stain over time. Our observation revealed high efficiency of the ABCB1-expressing melanoma cells to pump out Rhodamine 123. This activity could be inhibited with the p-gp pump inhibitor Elacridar (Fig. 3d). When treating the ABCB1-expressing cells with increasing concentration of karonudib, statistically significant less cytotoxic effect was observed for the ABCB1-expressing cells compared with the control cells (adjusted *p* value < 0.001). This protective effect could be reversed by using the p-gp pump inhibitor Elacridar (Fig. 3e). Finally, to assess if karonudib treatment affects *ABCB1* expression or causes selection of *ABCB1* high expressing cells, immunohistochemical staining of biopsies from four PDXes with high and one with low expression of *ABCB1* mRNA (Fig. 3a) was performed. Only one PDX (M150330) showed somewhat higher expression of ABCB1 after karonudib treatment, suggesting that this is not a common mechanism of acquired resistance (Fig. 1f). Taken together, the data show that high expression of p-gp pumps can make cells less sensitive to karonudib treatment.

### Karonudib treatment does not impair the T-cell-mediated anti-tumor response

**Fig. 4:**
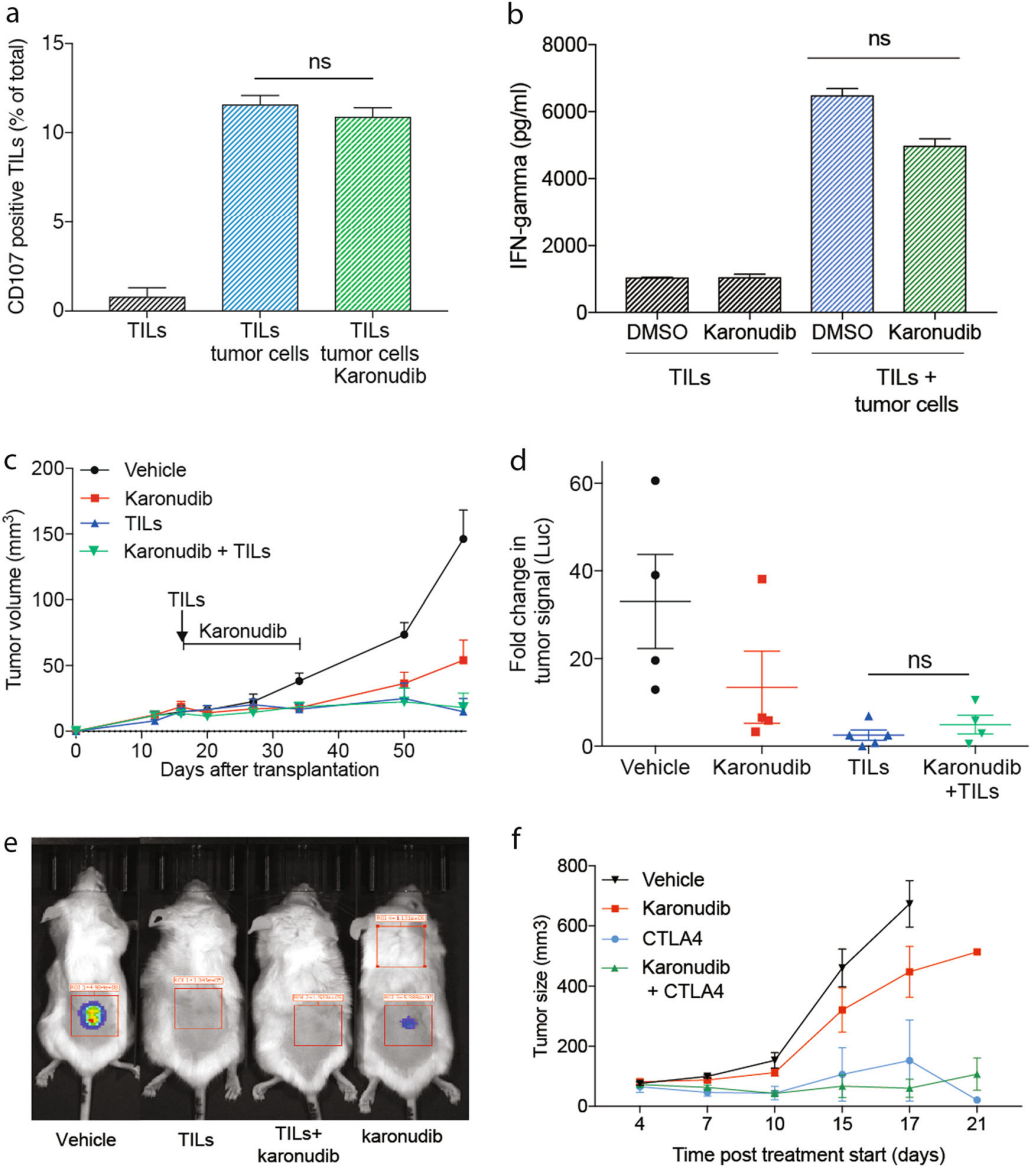
Karonudib treatment does not hamper T-cell-mediated anti-tumor immunity. **a** Quantitative analysis of the degranulation marker CD107 on TILs from patient sample M151002 grown with or without autologous tumor cells and with or without karonudib, data shown as average of triplicates (±SD). **b** Concentration of IFN-gamma in the media of the M15002 tumor cells cultured with or without autologous TILs and with or without karonudib. The ELISA data are shown as average of triplicates (±SD). **c**–**e** Size or luminescence signal of PDX tumors treated with vehicle (black), karonudib (red), injected with autologous TILs (blue) or karonudib + TILs (green), data are shown as average of four mice (±SEM). **f** B6 mice bearing tumors established from B16 mouse melanoma cell line, treated with either vehicle, CTLA4 antibody, karonudib, or combination of CTLA4 antibody and karonudib. Data in (**f**) shown as average tumor size of four mice (±SEM)

Immunotherapy has recently proven to be an efficient treatment for patients suffering from metastatic cutaneous melanoma. New targeted therapies or chemotherapies therefore need to spare the effector cells, primarily cytotoxic T cells, if used in combination with immunotherapy. To inform on the potential usefulness of karonudib in the immune therapy era, we investigated if karonudib would affect the anti-tumor activity of cytotoxic T cells. First, we expanded tumor-infiltrating T lymphocytes (TILs) from patient biopsy M151002 and then added them to short-term cultures of tumor cells from the same patient in the presence or absence of karonudib. This resulted in degranulation of the T cells, as measured by increased CD107 positive cells (Fig. 4a) and increased secretion of IFN-gamma (Fig. 4b) when TILs were co-cultured with autologous tumor cells for 24 h. Reassuringly, no significant decrease was observed when the cells were cultured in the presence of karonudib. To test if this would cause anti-tumor effects in vivo, we used our recently developed method to develop immune humanized PDX models (PDXv2)^9^. PDX-generated M151002 tumor cells were injected in NOG mice or NOG mice, transgenic for human IL2 (*hIL2*-NOG). When tumor growth was confirmed, we injected TILs in the *hIL2*-NOG mice, and subsequently treated half of the NOG mice and half of the *hIL2*-NOG mice with karonudib. Again, karonudib treatment did not impair the anti-tumor activity of TILs. The response was assessed by both measuring the physical size of the tumors (Fig. 4c) and the bioluminescence from the luciferase-expressing tumor cells (Fig. 4d, e). Furthermore, we tested if karonudib impaired the anti-tumor effect of anti-CTLA-4 treatment in immunocompetent mice bearing mouse melanoma tumors. We transplanted B16F10 mouse melanoma cells subcutaneously in B6 mice and treated with vehicle, anti-CTLA-4, karonudib, or combination of CTLA-4 and karonudib. Tumors on mice treated with anti-CTLA-4 antibody alone showed good responses by suppression of tumor growth, which was not impaired by the combination treatment with karonudib (Fig. 4f). Taken together, this indicates that karonudib does not have obvious negative effect on T-cell-mediated anti-tumor effect.

## Conclusion

Here we show that the previously published anti-cancer effects of karonudib in one melanoma PDX^4^ can be demonstrated in a larger cohort of animal models. Importantly though, we also demonstrate that some models remain unaffected by karonudib, suggesting resistance mechanisms. The data therefore confirm the utility of PDX trials when preclinically assessing small molecule inhibitor efficacy^10, 11^. In the clinic, the response evaluation criteria in solid tumors (RECIST) criterion is used to evaluate the treatment response^12^. In a recent PDX trial paper and in a second paper re-analyzing the same data, a modified RECIST criterion is used to define the response groups^10, 11^. When using the RECIST criterion, the inherent growth speed of the tumor is not taken into account when assessing the treatment response. Since we used a matching vehicle-treated PDX for all karonudib-treated PDXes, it allowed us to take the inherent tumor growth speed into consideration when categorizing samples in the response groups (treated/control). We observed that the tumors with the fastest inherent growth speed also exhibited the biggest size difference between the treated and the untreated tumors. Using normal RECIST, these tumors would have been categorized as stable disease. Our study shows that such a stable disease would have clear clinical benefit in comparison to no treatment.

Interestingly, we observe that karonudib has anti-tumor effect on melanoma PDXes, irrespective of mutation status of major melanoma driver genes. Earlier work suggested that oncogenic KRAS-driven tumors would benefit from MTH1 expression^13–15^. We did not see any difference in sensitivity between *BRAF*- and *NRAS*-mutated tumors. This either means that the same rules do not apply in melanoma, that oncogenic KRAS and NRAS differ or that the mechanism of action of karonudib extends outside MTH1 inhibition, e.g., the proposed microtubule disruption caused by inhibitors of the same class as karonudib^5–7^.

We also observed that four PDX models in the cohort exhibited a mutation or variant in *DDX3X*. The fact that all these models were in the regression group was interesting. DDX3X is an RNA-binding protein and when mutated, it is known to impair global translation and induce stress granulation assembly^16^. Stress granules are normally formed in cells under acute stress, for example, oxidative stress, UV irradiation, and heat shock. Furthermore, it has been shown that cells treated with microtubule-targeting agents show difference in size and location of stress granules^17^. However, we cannot, at this time, state that *DDX3X* mutation is a predictive biomarker due to two reasons: First, the functional analysis hampered by DDX3X knockdown was lethal and therefore only additive to the effects of karonudib. Second, none of the observed *DDX3X* mutations have been described before, meaning that additional analyses are needed to draw a firm conclusion.

One of the aims of this study was to identify a predictive biomarker of response. High expression of p-glycoprotein (p-pg) is a well-known drug-resistance mechanism^18^. P-gp pumps are transmembrane proteins, which act as ATP-dependent drug-efflux pumps. They are known to recognize and excrete compounds, even compounds the cell has not been exposed to before^19^. Furthermore, it has been shown that MDR genes such as *ABCB1* and *ABCG2* are dispensable for mouse development^20^, suggesting that they might be suitable targets. Here, we observe that high-expressing *ABCB1* cells are less sensitive to lower concentrations of karonudib than the control cells, which could be reversed by co-treating the cells with Elacridar (a known p-gp pump inhibitor^18^), establishing that karonudib is a p-gp substrate. That observation could be useful in the clinical setting for patients known to bear tumors with high p-gp pump expression, for example, in patients who have acquired resistance to chemotherapy^21^. Also, it raises the possibility of using p-gp pump inhibitors in combination with karonudib in the clinic. That combination treatment could be especially beneficial for melanoma patients, given the high expression of p-gp pumps in the blood–brain barrier, and that 10–20% of melanoma patients exhibit brain metastases.

It is likely that current treatment regiments only will cause durable responses or cures in subgroups of patients with melanoma. Current melanoma treatment includes both targeted therapy (BRAF and MEK inhibitors) as well as immune therapy (checkpoint block antibodies directed against PD1, PD-L1, and CTLA-4)^2^. Since durable responses to both these treatments have been correlated to abundance of tumor-infiltrating lymphocytes^22, 23^, it is imperative that novel treatments do not block the anti-tumor immunity. Hence, the fact that karonudib neither hinders adoptive T-cell transfer nor anti-CTLA-4 treatment in mice suggests that it can be used in combination with immune therapy.

Future use of karonudib is dependent on not only that it survives phase I clinical testing, but also that it causes clinically meaningful responses in patients. Most of the PDX models tested here reach a clinical response of stable disease. If this is reproduced in patients, combination treatments might be used to achieve tumor regression. Finding the right drug combinations (besides drug-pump inhibitors) is under active investigation.

## Materials and methods

### Ethical approvals

All experiments using patient material or research animals were performed according to the ethical approval provided by the Regional Human Ethics Board of Västra Götaland, Sweden #288-12 or by the Animal Ethics Board #2016-34, respectively.

### Patient material

All samples were obtained from patients who had provided informed consent and were treated at the Department of Surgery, Sahlgrenska University Hospital, Gothenburg, Sweden.

### Study design of the patient-derived xenograft clinical trial

Patient-derived xenografts were established from 31 metastatic melanoma patients, as previously described^8^. Each patient sample was transplanted in three mice. Palpable xenografts were measured three times weekly using a caliber and assigned to their treatment groups when they reached 50–100 mm^3^. The first mouse to reach the inclusion criterion was assigned to the karonudib treatment group, the second to the vehicle group, and the third mouse was excluded, utilizing the one mouse per patient per treatment group setup previously described^10^. Each mouse was treated for 18 days with either 90 mg/kg karonudib or the vehicle (20% w/v HPβCD in sodium acetate buffer pH 4.6) twice a day, three times per week. Treatment response was assessed by monitoring changes in tumor volume of the treated mouse and comparing to the tumor volume of the vehicle-treated mouse (treated/control (%)) using the following formulas:

$${\rm{tumor}}\,{\rm{volume}}\left( {{\rm{mm}}^3} \right)= \frac{{\rm{shorter}}\,{\rm{diameter}}^2\left( {\rm{mm}} \right) \times {\rm{longer}}\,{\rm{diameter}}{\rm{(mm)}}}{2}$$

and

$T{\mathrm{/}}C\left( {\mathrm{\% }} \right) = \frac{{T_i - T_0}}{{C_i - C_0}}$ where *T*_*i*_ and *C*_*i*_ represents tumor sizes at the end of the treatment and *T*_0_ and *C*_0_ represents tumor sizes at treatment start. Response criterion was as follows; progression if T/C > 50%, suppression if T/C = 50–0%, and regression if T/C < 0%. Weight of mice and tumor size were measured three times per week. After 18 days, mice were sacrificed and the tumors harvested and weighted. Tumor pieces were processed and preserved as snap-frozen or embedded in paraffin for RNA or protein analysis, respectively.

### RNA analysis

Tumors were dissociated using a Bullet Blender® and RNA was extracted using Nucleospin RNA kit (Machery-Nagel). Quantitative RNA analysis was conducted by synthesizing cDNA (iScript, BioRad) from the extracted RNA, which was quantified by qPCR (Kapa Biosystems). Primer sequences are available upon request. The extracted RNA was also used for RNA sequencing (SciLifeLabs NGS Core Facility, Stockholm, Sweden). Library was prepared using the Illumina TrueSeq with poly-A-selection and sequenced in a HiSeq 2500.

### Immunohistochemistry

Fresh tumor pieces were fixed in 4% formalin, dehydrated, and embedded in paraffin. Next, 5-μm sections were made, mounted on glass slides, and dried over night at 37 °C. Rehydration and antigen retrieval were performed by pressure cooking in citrate buffer. Staining was performed with an auto-stainer (Autostainer Link 48, Dako). Primary antibody staining was done for 60 min at room temperature, secondary for 20 min, and horseradish peroxidase staining for 20 min. DAB (Diaminobenzidine) staining was used to stain the DNA and counterstaining was done using hematoxylin. Finally, the sections were dehydrated, mounted with Pertex, and imaged.

### Antibodies

Anti-human Ki-67 antibody (DAKO) and anti-human ABCB1 antibody (Cell Signaling #13342).

### Compounds

Karonudib (TH1579) was synthesized at the Karolinska Institute according to previously published synthesis schemes (WO2015187088A1). Elacridar (GF120918) was bought from Selleck Chem (selleckchem.com).

### Bioinformatics

#### Alignment and pre-processing for variant calling

Raw reads were aligned to a combined human (hg19) and mouse (mm10) reference using STAR^24^ 2.5.2b with default parameters. The index file for STAR was augmented with splice junctions from a concatenated human GENCODE^25^ version 17 and mouse GENCODE version M7^26^ reference annotation. Reads mapping to human and mouse chromosomes, respectively, were then extracted using Samtools^27^ 0.1.19. For reads mapping to both organisms, Samtools and the FilterSamReads module of Picard 1.109 (*github.com/broadinstitute/picard*) were then used to discard those that were not primary alignments with respect to the human reference. This strategy was motivated by the desire to minimize false positives in variant calling due to potential mouse contamination in the tumor samples.

#### Variant calling and filtering

Single-nucleotide variants and indels were called by first using the mpileup module of Samtools with disabled BAQ computation, a supplied list of regions for the genes of interest as well as the hg19 reference. The resulting output was then supplied to the VarScan^28^ 2.3.9 tool mpileup2cns, for which the settings “min-coverage 2”, “min-reads2 2”, “min-var-freq 0.01”, and “min-avg-qual 15” were used. This very sensitive option was motivated by the desire to minimize the risk of false negatives owing to the lowly expressed genes and allelic imbalance, combined with the small list of genes being considered for calling. To further increase sensitivity, the two steps above were performed first on each sample individually, and then on a combination of all samples. The resulting call sets were then merged using the CombineVariants module of GATK^29^ 3.3.0.

Variant annotation was then performed using ANNOVAR^30^, cross-referencing the databases RefGene^31^, COSMIC^32^ v79 and dbSNP^33^ v138 with flagged somatic and clinically associated variants removed, ESP6500 (http://evs.gs.washington.edu/EVS), 1000 Genomes^34^ (August 2015). Synonymous variants, variants in non-exonic regions and those with an entry in the non-flagged dbSNP 138 database were then discarded. For increased specificity, only known COSMIC variants in oncogenes were retained. For tumor suppressors, non-COSMIC variants were also considered, provided that the variant was not present in the ESP6500 or 1000 Genomes databases. This was motivated by the fact that tumor suppressors are more likely to be subject to novel loss of function mutations. Additional manual inspection of variants was performed in IGV^35^, and novel mutations adjacent to homopolymer repeats or those only occurring at the ends of reads were discarded. Two additional potential mutations at known or recurrently mutated sites, although with very few supporting reads (2, 1, and 2 respectively), were added for samples M130111 (NRAS), M140602A (NRAS), and M120511B-2 (DDX3X) after visual inspection. The final set of mutations were visualized with Maftools^36^ R package.

#### Gene expression analysis

Differential expression analysis was performed in R, using the DESeq2^37^ package (default parameters), after first filtering out the unexpressed genes. Batch effects were accounted for in the DESeq2 regression model, since the samples M130128A, M130204B, M141204, M150313, and M160212 were sequenced at a different date. To visualize gene expression values, normalized and batch corrected counts were derived using the “removeBatchEffect” function in the limma^38^ package, based on values obtained with the “variance stabilizing transformation” DESeq2 method. Gene set enrichment analyses were performed on gene lists ranked by the log_2_-fold changes estimated by DESeq2 using FGSEA^39^, based on the MSigDB^40^ “Canonical pathways” gene set. A minimum gene set size of 25 and a maximum of 500 was specified and 1 million permutations were demanded.

### Cell lines

The melanoma cell lines SK-MEL-2 and MML-1 were purchased from the Cell Lines Service GmbH (Eppelheim, Germany), and HEK-293T was purchased from ATCC, Manassas, VA). They were cultured in 37 °C and 5% CO_2_ in the presence of DMEM-F12 (SK-MEL-2), RPMI1640 (MML-1), or DMEM media (HEK-293T) (Gibco), supplemented with 10% fetal bovine serum (Gibco) and Gentamycin (Thermo Fisher Scientific, Waltham, MA).

### Transfection

MML-1 cells were transfected with a pool of five different siRNAs against DDX3X (SMARTpool, Dharmacon) in the presence of DhermaFECT (Dharmacon).

### Cell cycle analysis

Harvested cells were re-suspended in modified Vindelövs solution (20 mM Tris, 100 mM NaCl, 1 µg/mL 7-aminoactinomycin D (7-AAD), 20 µg/mL RNase, and 0.1% nonidet P-40, adjusted to pH 8.0). The samples were analyzed using a BD Accuri C6 flow cytometer.

### Quantification of ABCB1 protein expression in vitro

Harvested cells were fixed and permeabilized (Fixation/Permeabilization solution, BD Bioscience). Next, the cells were stained with monoclonal rabbit ABCB1 antibody (Cell Signaling #13342) followed by staining with a secondary anti-Rabbit FITC (DAKO F0205). The stained cells were analyzed using a BD Accuri C6 flow cytometer.

### Analysis of p-gp pump activity

Cells were grown in the presence of 200 ng/mL Rhodamine 123 for 60 min with or without Elacridar (1 µM). After incubation, the cells were washed with PBS and cultured for another 90 min in fresh medium with or withour Elacridar (1 µM). Cell were harvested and re-suspended in PBS and analyzed with a BD Accuri C6 flow cytometer.

### Flow cytometry sorting of ABCB1 expressing cells

Cells were cultured in the presence of Rhodamine 123 (200 ng/mL) for 60 min. The harvested cells were sorted based on negative Rhodamine 123 signal, using Facs Aria IIIµ.

### Virus production and transduction

The calcium phosphate precipitation method was used to produce retroviruses by transfecting HEK-293T cells with the following plasmids; pHaMDRwt (kind gift from Michael Gottesman, Addgene plasmid #10957)^41^ and MSCV-IRES-PURO. Target cells were transduced over night and analyzed 72 h after transduction.

### Degranulation assay

TILs (tumor infiltrating lymphocytes) were extracted, cultured, and expanded in vitro by culturing primary tumor pieces in the presence of IL2. Next, the TILs and autologous tumor cells were incubated separately overnight with 0.5 µM karonudib or DMSO (1:20000), after which 3*10^5^ TILs and 1*10^5^ tumor cells were plated together in a 96 well V-bottom plate and cultured with monoclonal antibody for CD107a (LAMP-1)-APC human (clone: REA792) (Milteny Biotech). After the 6-h incubation, cells were washed once and re-suspended in MACS buffer before the surface expression of CD107 was determined by flow cytometry using a BD Accuri C6 flow cytometer.

### Measurement of IFN-gamma

IFN-gamma concentration in cell culture medium was measured using an enzyme-linked immunosorbent assay (ELISA) (Diaclone).

### PDXv2 mouse model

The method of making PDXv2 mouse models has been previously described^9^. Briefly, patient-derived tumor sample (M151002) was transduced with a pHAGE-luc-GFP virus and transplanted subcutaneously on the flanks of the immune deficient mice. Once tumor growth was confirmed by bioluminescence measurements (PerkinElmer IVIS Lumina III XR), the mice were separated in four treatment groups of four mice receiving either karonudib or vehicle (same concentrations and dosing schedule as in the pre-clinical PDX trial) with or without TILs (one injection of 20*10^6^ TILs). Tumor growth was followed by caliper and bioluminescence measurements.

### Statistical analysis

Graphs and statistical testing were generated using GraphPad Prism, error bars on growth curves are shown as standard error of mean (SEM), and other error bars are shown as Standard error of mean (SD). Student’s *t* test was performed, where statistically significant *P* value was indicated as; *P < 0.05, **P < 0.01, ***P < 0.001, or ****P < 0.0001.

## Electronic supplementary material

Supplemental figure 1

Supplemental figure 2

Supplemental figure 3

Supplemental figure 4

Supplemental figure 5

Supplemental table 1

Supplemental table 2

Supplemental table 3

Supplemental table 4

Supplementary figure legends

## References

[1] Garbe C (2016). Diagnosis and treatment of melanoma. European consensus-based interdisciplinary guideline - update 2016. Eur. J. Cancer.

[2] Ugurel S (2017). Survival of patients with advanced metastatic melanoma: the impact of novel therapies-update 2017. Eur. J. Cancer.

[3] Gad H (2014). MTH1 inhibition eradicates cancer by preventing sanitation of the dNTP pool. Nature.

[4] Warpman Berglund U (2016). Validation and development of MTH1 inhibitors for treatment of cancer. Ann. Oncol..

[5] Kawamura T (2016). Proteomic profiling of small-molecule inhibitors reveals dispensability of MTH1 for cancer cell survival. Sci. Rep..

[6] Kettle JG (2016). Potent and selective inhibitors of MTH1 probe its role in cancer cell survival. J. Med. Chem..

[7] Wang JY (2016). Reactive oxygen species dictate the apoptotic response of melanoma cells to TH588. J. Invest. Dermatol..

[8] Einarsdottir BO (2014). Melanoma patient-derived xenografts accurately model the disease and develop fast enough to guide treatment decisions. Oncotarget.

[9] Jespersen H (2017). Clinical responses to adoptive T-cell transfer can be modeled in an autologous immune-humanized mouse model. Nat. Commun..

[10] Gao H (2015). High-throughput screening using patient-derived tumor xenografts to predict clinical trial drug response. Nat. Med..

[11] Ben-David U (2017). Patient-derived xenografts undergo mouse-specific tumor evolution. Nat. Genet..

[12] Eisenhauer EA (2009). New response evaluation criteria in solid tumours: revised RECIST guideline (version 1.1). Eur. J. Cancer.

[13] Patel A (2015). MutT Homolog 1 (MTH1) maintains multiple KRAS-driven pro-malignant pathways. Oncogene.

[14] Rai P (2009). Continuous elimination of oxidized nucleotides is necessary to prevent rapid onset of cellular senescence. Proc. Natl Acad. Sci. USA.

[15] Rai P (2011). Enhanced elimination of oxidized guanine nucleotides inhibits oncogenic RAS-induced DNA damage and premature senescence. Oncogene.

[16] Valentin-Vega YA (2016). Cancer-associated DDX3X mutations drive stress granule assembly and impair global translation. Sci. Rep..

[17] Chernov KG (2009). Role of microtubules in stress granule assembly: microtubule dynamical instability favors the formation of micrometric stress granules in cells. J. Biol. Chem..

[18] Binkhathlan Z, Lavasanifar A (2013). P-glycoprotein inhibition as a therapeutic approach for overcoming multidrug resistance in cancer: current status and future perspectives. Curr. Cancer Drug Targets.

[19] Kunjachan S, Rychlik B, Storm G, Kiessling F, Lammers T (2013). Multidrug resistance: physiological principles and nanomedical solutions. Adv. Drug Deliv. Rev..

[20] Schinkel AH (1994). Disruption of the mouse mdr1a P-glycoprotein gene leads to a deficiency in the blood-brain barrier and to increased sensitivity to drugs. Cell.

[21] Leslie EM, Deeley RG, Cole SP (2005). Multidrug resistance proteins: role of P-glycoprotein, MRP1, MRP2, and BCRP (ABCG2) in tissue defense. Toxicol. Appl. Pharmacol..

[22] Wilmott JS (2012). Selective BRAF inhibitors induce marked T-cell infiltration into human metastatic melanoma. Clin. Cancer Res..

[23] Taube JM (2014). Association of PD-1, PD-1 ligands, and other features of the tumor immune microenvironment with response to anti-PD-1 therapy. Clin. Cancer Res..

[24] Dobin A (2013). STAR: ultrafast universal RNA-seq aligner. Bioinformatics.

[25] Harrow J (2012). GENCODE: the reference human genome annotation for the ENCODE project. Genome Res..

[26] Mudge JM, Harrow J (2015). Creating reference gene annotation for the mouse C57BL6/J genome assembly. Mamm. Genome.

[27] Li H (2009). The sequence alignment/map format and SAMtools. Bioinformatics.

[28] Koboldt DC (2012). VarScan 2: somatic mutation and copy number alteration discovery in cancer by exome sequencing. Genome Res..

[29] Warden CD, Adamson AW, Neuhausen SL, Wu X (2014). Detailed comparison of two popular variant calling packages for exome and targeted exon studies. PeerJ.

[30] Wang K, Li M, Hakonarson H (2010). ANNOVAR: functional annotation of genetic variants from high-throughput sequencing data. Nucleic Acids Res..

[31] O’Leary NA (2016). Reference sequence (RefSeq) database at NCBI: current status, taxonomic expansion, and functional annotation. Nucleic Acids Res..

[32] Forbes Sa (2011). COSMIC: mining complete cancer genomes in the catalogue of somatic mutations in cancer. Nucleic Acids Res..

[33] Sherry ST (2001). dbSNP: the NCBI database of genetic variation. Nucleic Acids Res..

[34] The Genomes Project, C., Vol. **526** 68–74 (2015).

[35] Thorvaldsdóttir H, Robinson JT, Mesirov JP (2013). Integrative Genomics Viewer (IGV): high-performance genomics data visualization and exploration. Brief Bioinform..

[36] Mayakonda, A. & Koeffler, H. P. Maftools: efficient analysis, visualization and summarization of MAF files from large-scale cohort based cancer studies. *bioRxiv*10.1101/052662 (2016).

[37] Love MI, Huber W, Anders S (2014). Moderated estimation of fold change and dispersion for RNA-seq data with DESeq2. Genome Biol..

[38] Ritchie ME (2015). limma powers differential expression analyses for RNA-sequencing and microarray studies. Nucleic Acids Res..

[39] Sergushichev, A. An algorithm for fast preranked gene set enrichment analysis using cumulative statistic calculation. *bioRxiv*10.1101/060012 (2016).

[40] Subramanian A., Tamayo P., Mootha V. K., Mukherjee S., Ebert B. L., Gillette M. A., Paulovich A., Pomeroy S. L., Golub T. R., Lander E. S., Mesirov J. P. (2005). Gene set enrichment analysis: A knowledge-based approach for interpreting genome-wide expression profiles. Proceedings of the National Academy of Sciences.

[41] Pastan I (1988). A retrovirus carrying an MDR1 cDNA confers multidrug resistance and polarized expression of P-glycoprotein in MDCK cells. Proc. Natl Acad. Sci. USA.

