# Correction: A patient-derived xenograft pre-clinical trial reveals treatment responses and a resistance mechanism to karonudib in metastatic melanoma

**DOI:** 10.1038/s41419-020-2301-y

**Published:** 2020-02-06

**Authors:** Berglind O. Einarsdottir, Joakim Karlsson, Elin M. V. Söderberg, Mattias F. Lindberg, Elisa Funck-Brentano, Henrik Jespersen, Siggeir F. Brynjolfsson, Roger Olofsson Bagge, Louise Carstam, Martin Scobie, Tobias Koolmeister, Olof Wallner, Ulrika Stierner, Ulrika Warpman Berglund, Lars Ny, Lisa M. Nilsson, Erik Larsson, Thomas Helleday, Jonas A. Nilsson

**Affiliations:** 1 Sahlgrenska Translational Melanoma Group, Sahlgrenska Cancer Center, Departments of Surgery and Oncology, Institute of Clinical Sciences, University of Gothenburg and Sahlgrenska University Hospital, Gothenburg, Sweden; 2 Department of Medical Chemistry, Institute of Biomedicine, University of Gothenburg, Gothenburg, Sweden; 3 Department of Microbiology and Immunology, Institute for Biomedicine, Sahlgrenska Academy, University of Gothenburg, Gothenburg, Sweden; 4 Department of Neurosurgery, Sahlgrenska University Hospital, Gothenburg, Sweden; 5 Science for Life Laboratory, Division of Translational Medicine and Chemical Biology, Department of Medical Biochemistry and Biophysics, Karolinska Institutet, Stockholm, Sweden

**Correction to: Cell Death and Disease**

10.1038/s41419-018-0865-6, published online 24 July 2018

On Pubmed, the name of co-author Roger Olofsson Bagge appeared incorrectly as “Bagge RO” instead of “Olofsson Bagge, Roger”.

This has been corrected in the PDF and HTML versions.

