# Supplementary figures and images for: A patient-derived xenograft pre-clinical trial reveals treatment responses and a resistance mechanism to karonudib in metastatic melanoma
Source: Cell Death Dis. 2018 Jul 24;9(8):810. doi: 10.1038/s41419-018-0865-6 (PMC6057880; doi:10.1038/s41419-018-0865-6)

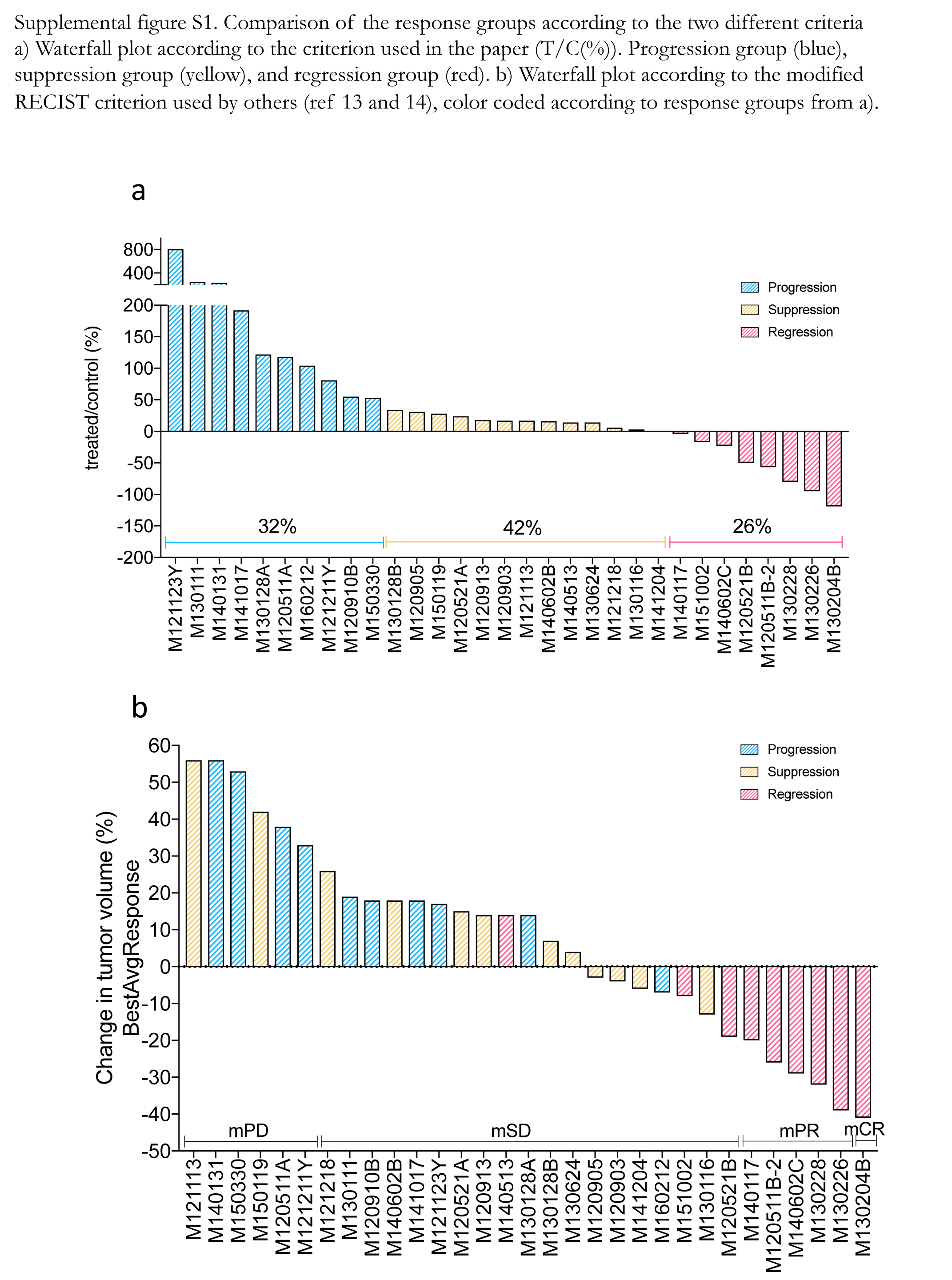

Supplement: Supplementary file 1 — Supplemental figure 1 [file 41419_2018_865_MOESM1_ESM.tif]

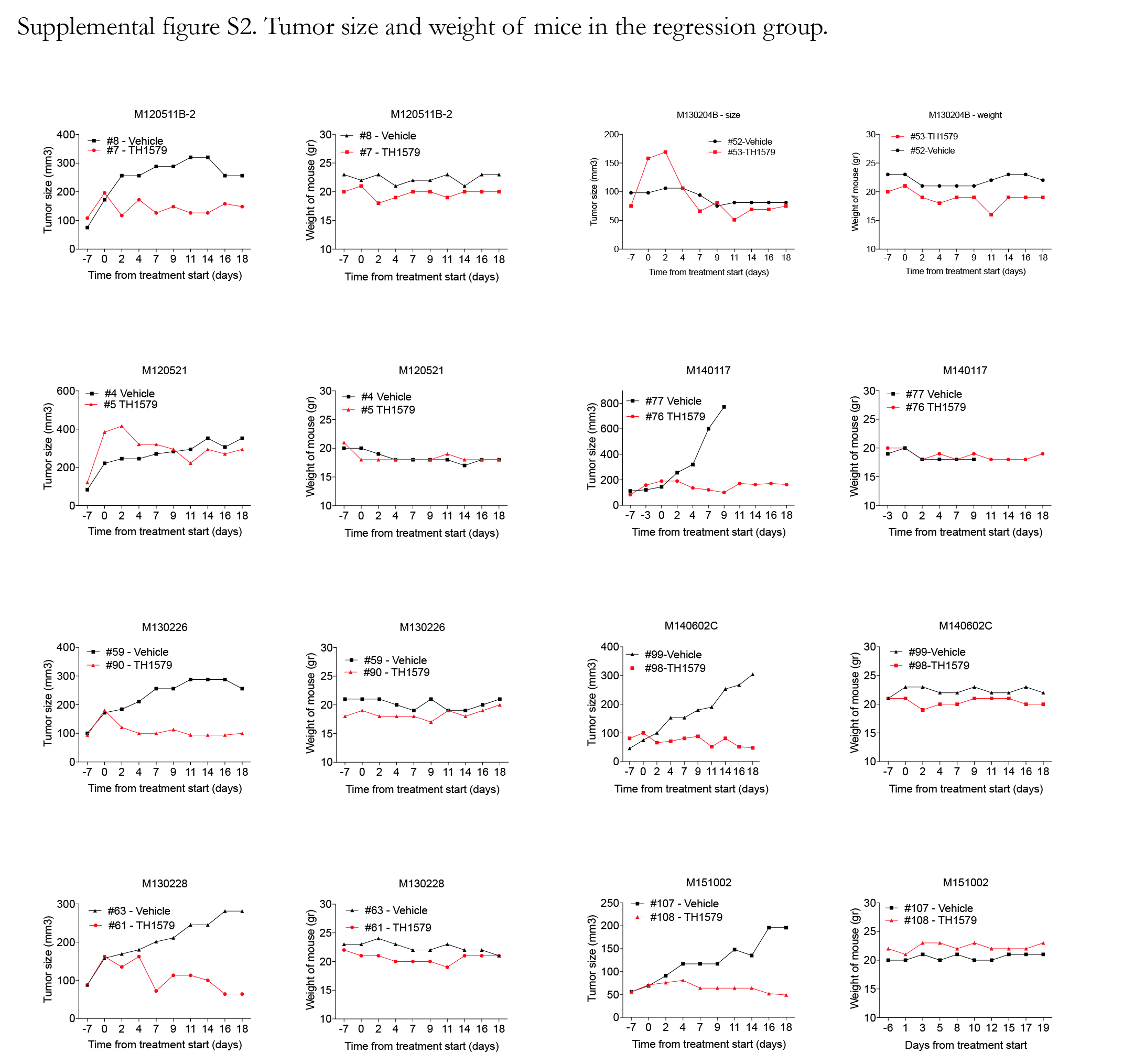

Supplement: Supplementary file 2 — Supplemental figure 2 [file 41419_2018_865_MOESM2_ESM.tif]

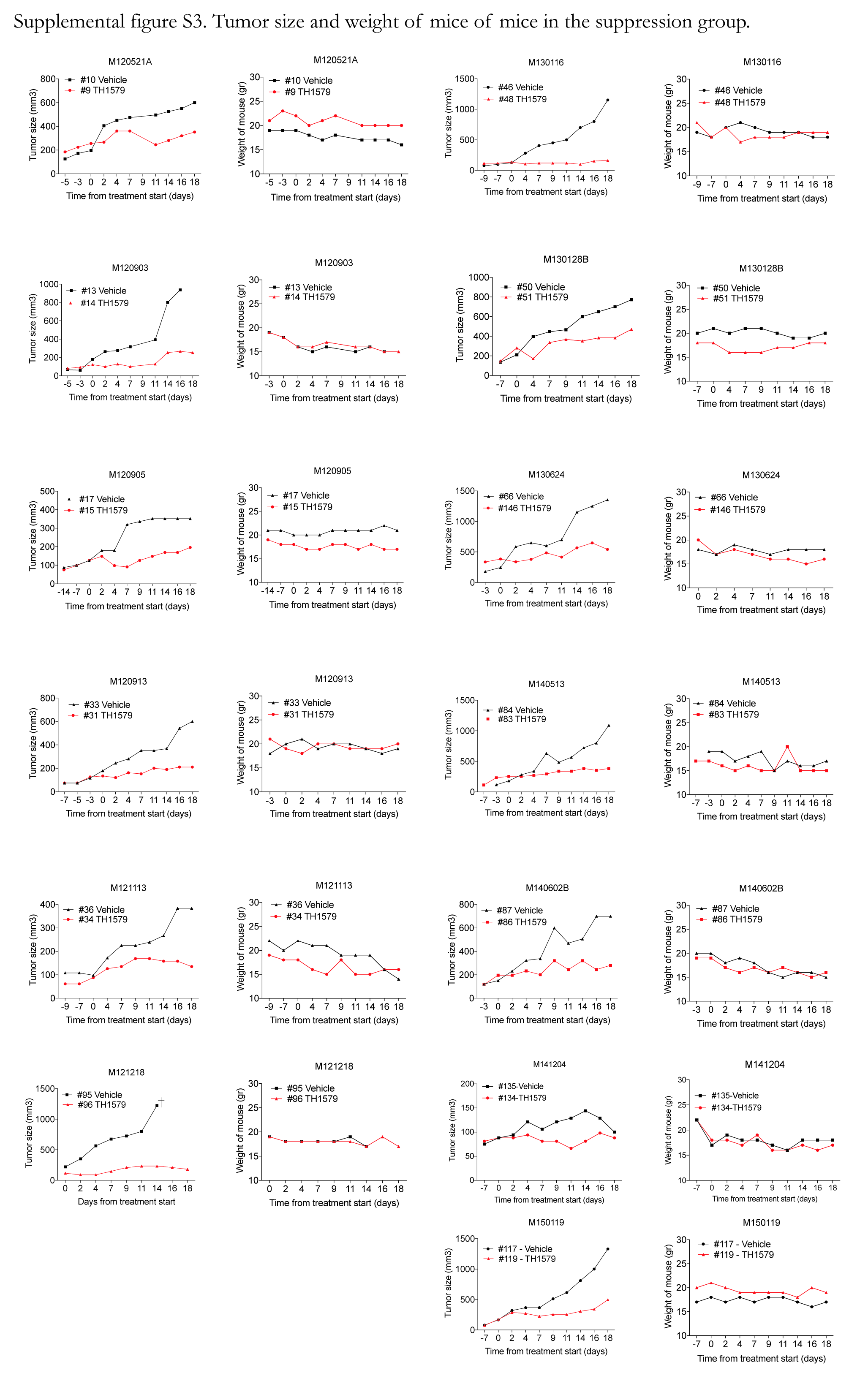

Supplement: Supplementary file 3 — Supplemental figure 3 [file 41419_2018_865_MOESM3_ESM.tif]

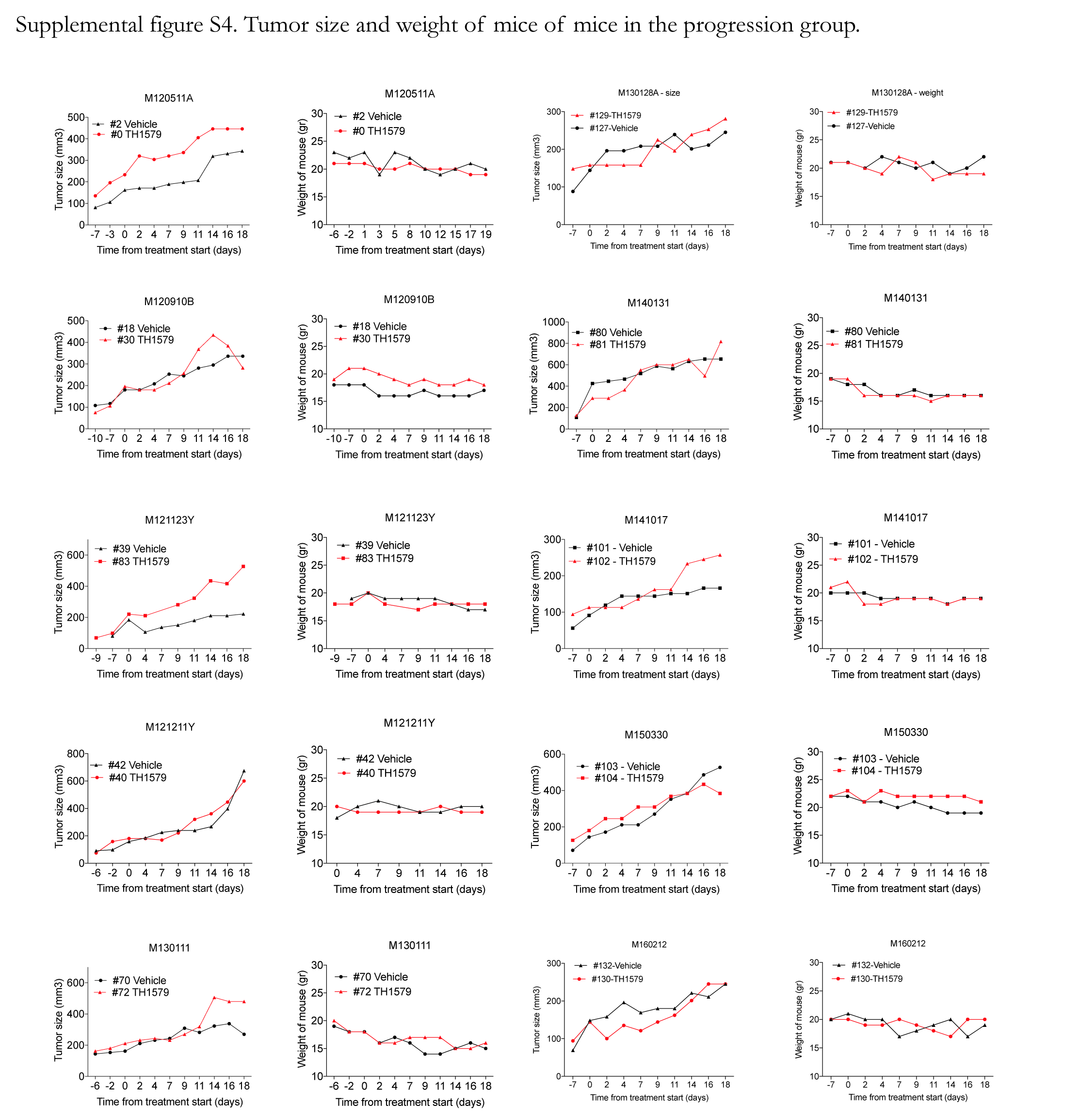

Supplement: Supplementary file 4 — Supplemental figure 4 [file 41419_2018_865_MOESM4_ESM.tif]

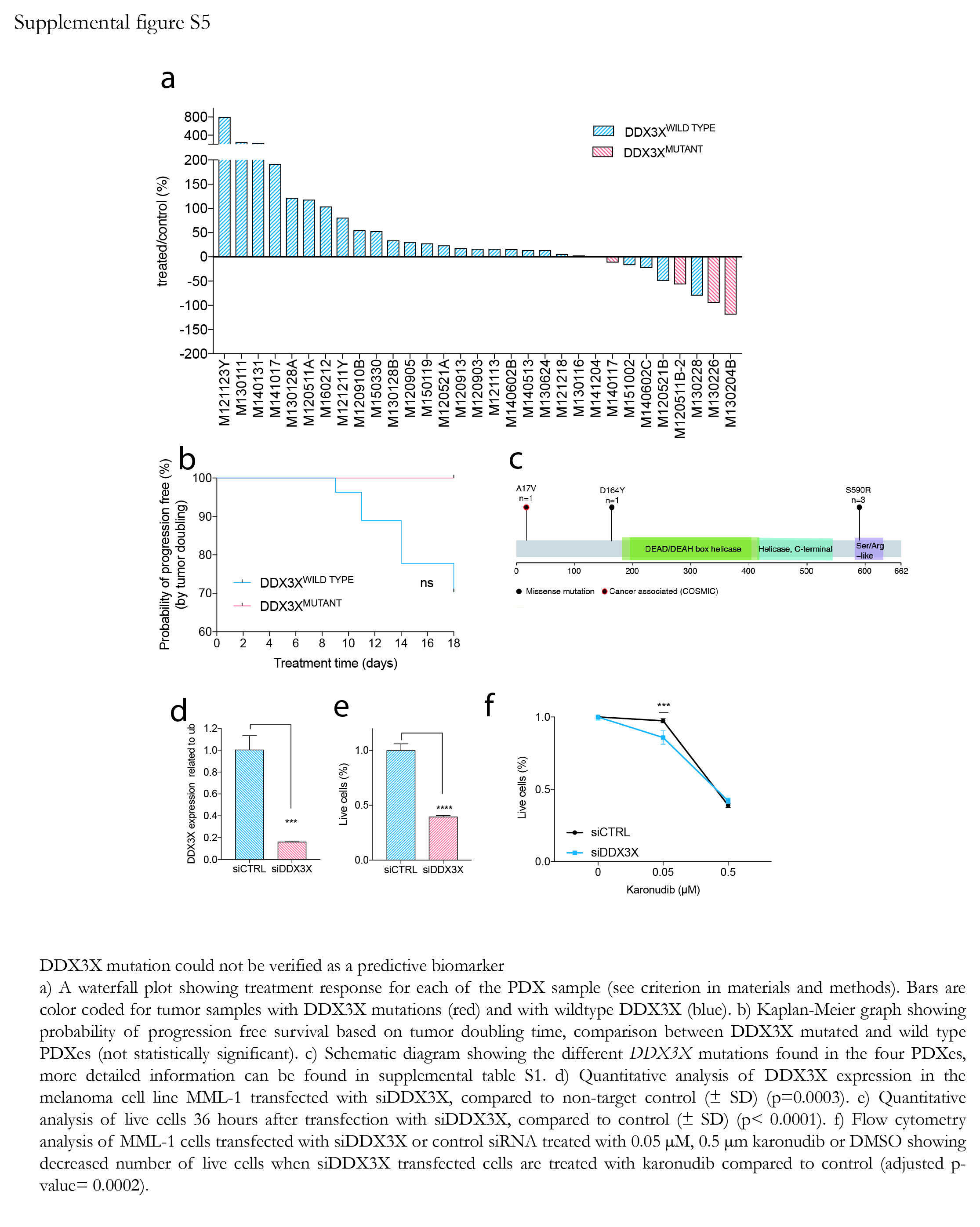

Supplement: Supplementary file 5 — Supplemental figure 5 [file 41419_2018_865_MOESM5_ESM.tif]
