# Supplementary material for: A patient-derived xenograft pre-clinical trial reveals treatment responses and a resistance mechanism to karonudib in metastatic melanoma
Source: Cell Death Dis. 2018 Jul 24;9(8):810. doi: 10.1038/s41419-018-0865-6 (PMC6057880; doi:10.1038/s41419-018-0865-6)
Supplement: Supplementary file 6 — Supplemental table 1 [file 41419_2018_865_MOESM6_ESM.pdf]

Supplemental table 1. Patient and tumor biopsy information

	Patient ID	YOB	Gender	Genotype	Stage of disease	Treatment before biopsy
Progression group	M120511A	1961	Male	BRAF mut	IV	BRAFi
	M120910B	1933	Male	NRAS mut	IV	No treatment
	M121123Y	1931	Female	BRAF mut	IIIC	ILP
	M121211Y	1932	Male	BRAF mut	IIIC	No treatment
	M130111	1960	Male	NRAS mut	IIIC	No treatment
	M130128A	1944	Male	NRAS mut	IV	No treatment
	M140131	1954	Female	BRAF mut	IV	No treatment
	M141017	1973	Female	BRAF mut	IV	Carboplatin+DTIC, Eldisine, Ipilimumab, Paclitaxel, BRAFi
	M150330	1946	Female	BRAF mut	IV	BRAFi
	M160212	1954	Male	NRAS mut	IV	No treatment
Suppression group	M120521A	1953	Male	BRAF mut	IV	No treatment
	M120903	1955	Male	NRAS mut	IIIC	No treatment
	M120905	1952	Male	wt	IIIC	No treatment
	M120913	1936	Male	wt	IV	Temozolomide, Eldisine
	M121113	1972	Female	BRAF mut	IIIC	No treatment
	M121218	1947	Male	BRAF mut	IV	DTIC+radio therapy, Carboplatin, BRAFi, Paclitaxel, Eldesin
	M130116	1975	Female	BRAF mut	IV	No treatment
	M130128B	1939	Male	NRAS mut	IV	No treatment
	M130624	1972	Female	BRAF mut	IV	No treatment
	M140513	1973	Female	BRAF mut	IV	Carboplatin+DTIC, Eldisine, Ipilimumab, Paclitaxel, BRAFi
	M140602B	1954	Female	BRAFmut,NRAS mut	IV	BRAFi
	M141204	1949	Male	BRAF mut	IV	BRAFi, ipilimumab
	M150119	1975	Female	BRAF mut	IV	BRAFi+MEKi, ipilimumab
Regression group	M120511B-2	1959	Male	BRAF mut	IV	BRAFi, Temozolomide, Cisplatin
	M120521B	1937	Female	NRAS mut	IV	No treatment
	M130204B	1934	Male	BRAF mut	IIIC	No treatment
	M130226	1951	Female	BRAF mut	IV	MEKi
	M130228	1945	Male	NRAS mut	IIIC	ILP
	M140117	1939	Female	BRAFmut,NRAS mut	IV	Temozolomide
	M140602C	1957	Female	BRAF mut	IV	BRAFi+MEKi
	M151002	1954	Male	NRAS mut	IV	No treatment
