# Supplementary material for: A patient-derived xenograft pre-clinical trial reveals treatment responses and a resistance mechanism to karonudib in metastatic melanoma
Source: Cell Death Dis. 2018 Jul 24;9(8):810. doi: 10.1038/s41419-018-0865-6 (PMC6057880; doi:10.1038/s41419-018-0865-6)
Supplement: Supplementary file 7 — Supplemental table 2 [file 41419_2018_865_MOESM7_ESM.pdf]

Supplemental table 2. Missense DDX3X mutations in the PDXes used in the pre-clinical trial

Sample ID	Chr.	Start Position	End Position	Strand	HGVSc	HGVSp
M140117	chrX	41196665	41196665	+	c.50N>T	p.Ala17Val
M140117	chrX	41206565	41206565	+	c.1770N>G	p.Ser590Arg
M120511B-2	chrX	41206565	41206565	+	c.1770N>G	p.Ser590Arg
M130226	chrX	41202036	41202036	+	c.490N>T	p.Asp164Tyr
M130204B	chrX	41206565	41206565	+	c.1770N>G	p.Ser590Arg
