# Supplementary material for: A patient-derived xenograft pre-clinical trial reveals treatment responses and a resistance mechanism to karonudib in metastatic melanoma
Source: Cell Death Dis. 2018 Jul 24;9(8):810. doi: 10.1038/s41419-018-0865-6 (PMC6057880; doi:10.1038/s41419-018-0865-6)
Supplement: Supplementary file 8 — Supplemental table 3 [file 41419_2018_865_MOESM8_ESM.pdf]

Supplemental table S3. Total number of read pairs obtained for each sample.

PatientID	Reads
M121211	41735449
M130624	32974641
M120903	27503725
M120521A	17052687
M120913	25478941
M121113	29439383
M121123Y	37387624
M130116	40138455
M130128B	31907691
M120511A	30041463
M130111	27517795
M140117	40503607
M140513	40063463
M140602B	40404327
M120910B	39344988
M140131	47992689
M120521B	44599800
M121218	49255334
M120905	40163976
M120511B-2	43187744
M130226	39466003
M130228	32712504
M141017	42204419
M150119	34159560
M150330	40271536
M151002	40566447
M140602C	29405720
M130128A	60951617
M130204B	50819668
M141204	49333767
M160212	47324800
