# Supplementary material for: A patient-derived xenograft pre-clinical trial reveals treatment responses and a resistance mechanism to karonudib in metastatic melanoma
Source: Cell Death Dis. 2018 Jul 24;9(8):810. doi: 10.1038/s41419-018-0865-6 (PMC6057880; doi:10.1038/s41419-018-0865-6)
Supplement: Supplementary file 10 — Supplementary figure legends [file 41419_2018_865_MOESM10_ESM.docx]

Supplemental figure S1. Comparison of the response groups according to the two different criteria. a) Waterfall plot according to the criterion used in the paper (T/C(%)). Progression group (blue), suppression group (yellow), and regression group (red). b) Waterfall plot according to the modified RECIST criterion used by others (ref 10 and 11), color coded according to response groups from a).

Supplemental figure S2. Tumor size and weight of mice in the regression group.

Supplemental figure S3. Tumor size and weight of mice in the suppression group.

Supplemental figure S4. Tumor size and weight of mice in the progression group.

Supplemental figure S5. DDX3X mutation could not be verified as a predictive biomarker.

a) A waterfall plot showing treatment responses for each of the PDX sample (see criterion in materials and methods). Bars are color coded for tumor samples with DDX3X mutations (red) and with wildtype DDX3X (blue). b) Kaplan-Meier graph showing probability of progression free survival based on tumor doubling time, comparison between DDX3X mutated and wild type PDXes (not statistically significant). c) Schematic diagram showing the different *DDX3X* mutations found in the four PDXes, more detailed information can be found in supplemental table S1. d) Quantitative analysis of DDX3X expression in the melanoma cell line MML-1 transfected with siDDX3X, compared to control (± SD) (p< 0.0001). f) Flow cytometry analysis of MML-1 cells transfected with siDDX3X or control siRNA treated with 0.05 µM, 0.5 µM karonudib or DMSO showing decreased number of live cells when siDDX3X transfected cells are treated with karonudib compared to control. (adjusted p-value = 0.0002).

Supplemental table 1. Patient and tumor biopsy information

Supplemental table 2. Missense DDX3X mutations in the PDXes used in the pre-clinical trial

Supplemental table 3. Total number of read pairs obtained for each sample

Supplemental table 4. Differentially expressed genes between the response groups
